# Ubiquitin beyond the proteome: lipids, glycans, metabolites, nucleic acids, and an expanding molecular landscape

**DOI:** 10.1042/BST20260081

**Published:** 2026-06-12

**Authors:** Akinori Endo, Yukiko Yoshida

**Affiliations:** Laboratory of Protein Metabolism, Tokyo Metropolitan Institute of Medical Science, Tokyo 156-8506, Japan

**Keywords:** non-proteinaceous, Ubiquitin, Ubiquitin ligases

## Abstract

Ubiquitination is a versatile post-translational modification process in which the small globular protein ubiquitin is covalently attached to substrate proteins to generate diverse cellular signals. Although originally characterized by its role in proteasome-mediated protein degradation, ubiquitination is now recognized as a central regulator of numerous processes, including signaling, trafficking, and immunity. Canonical ubiquitination is mediated by a cascade of E1 (activating), E2 (conjugating), and E3 (ligase) enzymes that repeatedly conjugate ubiquitin molecules to lysine residues on substrate proteins, leading to the formation of polyubiquitin chains with distinct topologies. The modification is reversed by deubiquitinating enzymes. Notably, components of the ubiquitin system comprise approximately 7% of the human proteome, underscoring its importance in biological regulation. Recent advances have revealed the broad scope of ubiquitination. Ubiquitin was found to conjugate not only to lysine but also to serine, threonine, and cysteine, indicating its unexpected chemical flexibility. Furthermore, ubiquitination can be directed toward other post-translational modifications, particularly glycosylation and ADP-ribosylation, highlighting the extensive crosstalk between modification systems. Strikingly, lipids, sugars, metabolites, nucleic acids, and even synthetic small-molecule compounds have been identified as ubiquitinated substrates. The hypothesis that virtually all classes of molecules are targeted by ubiquitination has become increasingly plausible. Taken together, these findings redefine ubiquitination as a far more general modification process than previously appreciated. In this mini-review, we focus on recent progress in non-proteinaceous ubiquitination research, summarize emerging substrate classes, and discuss key challenges in elucidating the underlying mechanisms and physiological roles of this expanding modification landscape.

## Introduction

**Figure 1:**
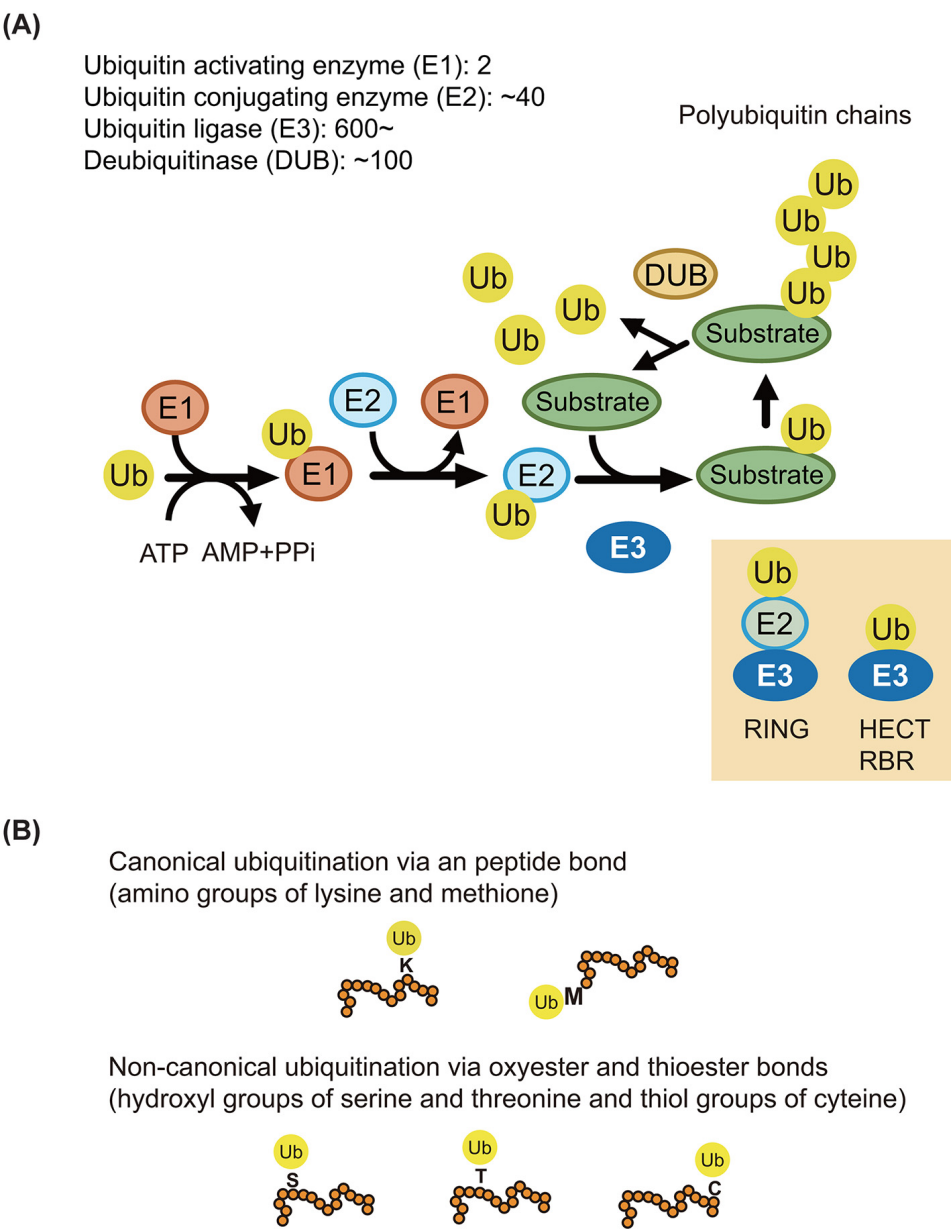
Proteinaceous ubiquitination (**A**) Schematic diagram of the Ub system. Ub is activated by E1 in an ATP-dependent reaction and forms a thioester adduct with the enzyme. Ub is transferred to E2 in a transthiolation reaction. RING-type E3 ligases transfer Ub from E2 directly to substrates, whereas HECT- and RBR-type E3 ligases first covalently bind Ub at their active-site cysteine residue before transferring it to substrates. This reaction can be repeated using either a lysine residue or the N-terminal methionine (M1) of Ub for attachment of the next Ub molecule, giving rise to polyUb chains. Ubiquitination can be reversed by deubiquitinating enzymes (DUBs). (**B**) Ubiquitination on amino acid residues of protein substrates. Canonical ubiquitination occurs through the formation of the most stable amide bound, in which the C-terminus of Ub forms either an isopeptide bond with the ε-amino group of a lysine residue in the substrate protein or a peptide bond with the α-amino group of its M1 residue. By contrast, non-canonical ubiquitination includes oxyester bond formation between the C-terminus of Ub and the hydroxyl group of serine or threonine as well as thioester bond formation with the thiol group of cysteine. Compared with the amide bond, the thioester bond is the most reactive and therefore the least stable, whereas the oxyester bond occupies an intermediate position in reactivity.

Among the various post-translational modifications (PTMs), ubiquitination is unique in that the modifier is a small protein, ubiquitin (Ub). Ubiquitination proceeds via a cascade of three classes of enzymes: Ub-activating enzymes (E1), Ub-conjugating enzymes (E2), and Ub ligases (E3). E1 enzymes activate Ub in an ATP-dependent manner, transferring it to E2 enzymes [1,2]. E3 ligases recognize specific substrates and mediate the transfer of Ub to them either from the E2 enzyme directly, as executed by really interesting new gene (RING)-type ligases, or via a thioester intermediate on the E3 ligase itself, as performed by homologous to the E6-AP carboxyl terminus (HECT)-type and RING-in-between-RING (RBR)-type ligases (Figure 1A) [3,4]. In addition to these canonical enzyme classes, atypical E3 ligases that are not categorized as RING, HECT, or RBR types have also been identified. A recent systematic characterization of the E3 ligase landscape identified 672 high-confidence members of the class encoded by the human genome [5]. More broadly, a comprehensive survey of the Ub-proteasome system has estimated that it comprises over 1400 distinct proteins in humans [6], representing approximately 7% of the ∼20,000 proteins-coding genes in the human genome. This remarkable complexity underlies the diverse roles of this system in proteostasis, encompassing not only protein quality control but also a wide array of regulatory functions.

In the canonical reaction, the C-terminal glycine of Ub forms an isopeptide bond with the ε-amino group of lysine residues in substrate proteins [1,2]. Ub itself is ubiquitinated at any of its seven lysine residues (K6, K11, K27, K29, K33, K48, and K63) or N-terminal methionine (M1) to form polyUb chains [7]. Among these linkage types, K48-linked Ub chains are the most abundant and serve as proteolytic signals for proteasomal degradation, thereby directing the removal of misfolded, damaged, or superfluous proteins. By contrast, K63-linked Ub chains primarily mediate non-proteolytic signaling and participate in diverse cellular processes, including endocytosis, selective autophagy, DNA damage responses, and organelle stress signaling. Additional regulatory complexity arises from the other PTMs of Ub, including its phosphorylation and acetylation, constituting a layer of regulation that is made possible because the modifier is a protein. These combinatorial Ub codes are interpreted by reader proteins containing specific Ub-binding domains that enable Ub signals to be decoded into diverse downstream outcomes. Similar to many PTMs, ubiquitination is reversible and dynamically regulated by DUBs, which remove Ub from substrates and remodel Ub chains (reviewed in [8–12]).

As mentioned above, canonical protein ubiquitination targets lysine residues via isopeptide bonds. However, the targeting of other amino acids via ester bonds in non-canonical protein ubiquitination has also been reported (Figure 1B). For example, in endoplasmic reticulum-associated degradation (ERAD), the oxyester bonds formed during serine- and threonine-directed ubiquitination are catalyzed by certain viral E3 ligases that co-opt the ERAD pathway [13,14], and by the host ERAD-E3 ligase Hrd1 [15,16]. Within the linear Ub chain assembly complex (LUBAC), which comprises HOIP, HOIL-1, and SHARPIN components, HOIP catalyzes the formation of M1-linked linear Ub chains on substrates such as nuclear factor kappa B (NF-κB) essential modulator and receptor-interacting protein kinase 1, which are central mediators of NF-κB signaling and inflammatory responses [17–19]. By contrast, HOIL-1 ubiquitinates serine or threonine residues on interleukin receptor-associated kinase (IRAK) 1, IRAK2, and IRAK4 within the myddosome, thereby contributing to the activation of Toll-like receptor signaling pathways [20,21]. MYC-binding protein 2 (MYCBP2), a RING-Cys-relay E3 ligase, coordinates the thioester and oxyester bonds of Ub. In brief, the distinctive tandem cysteine catalytic domain of MYCBP2 both relays and conjugates Ub to threonine residues within the hydrophobic pocket of the substrate [22]. This activity promotes the destabilization and degradation of nicotinamide mononucleotide adenylyltransferase 2 (NMNAT2), thereby influencing axonal degeneration and neuronal fate [22,23]. In addition to serine and threonine ubiquitination, the monoubiquitination of peroxisomal biogenesis factor 5 (PEX5) at cysteine (C11) via a thioester bond plays a crucial role in the import of cargo proteins to peroxisomes during biogenesis [24–26]. Cysteine ubiquitination has also been described in studies on the quality control of peroxisomal proteins [25,27,28]. Collectively, these findings establish non-lysine ubiquitination as an emerging and functionally important layer of regulatory complexity. Similar to canonical ubiquitination, non-lysine ubiquitination appears to be reversible [29]. Although the implicated DUBs and their physiological substrates remain incompletely characterized, several classes of DUB have been established to have Ub oxyesterase and thioesterase activities [30,31].

**Figure 2:**
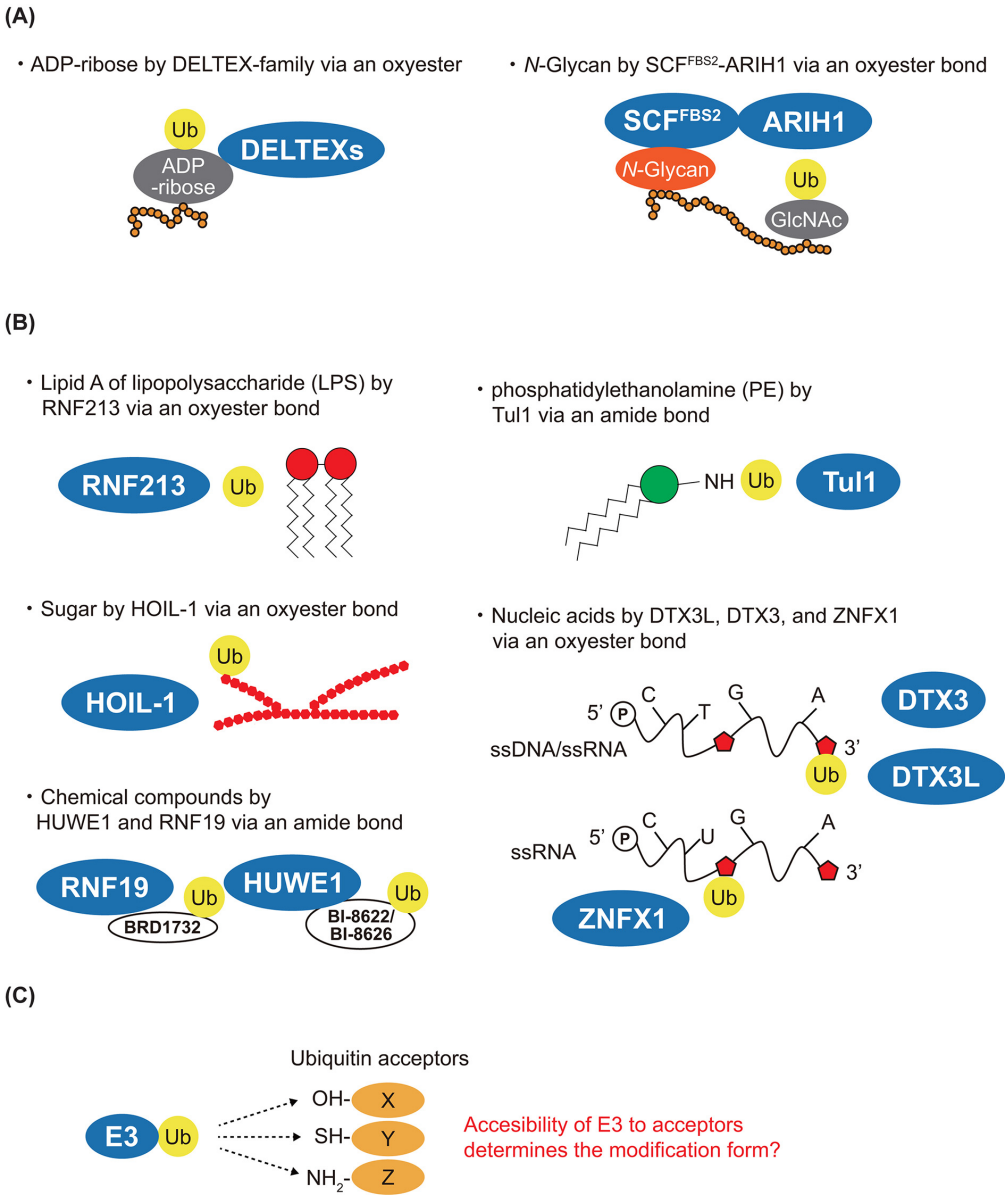
Non-proteinaceous ubiquitination discussed in this review (**A**) Ubiquitination of PTM adducts. DELTEX-family E3 ligases mediate oxyester bond formation between the 3′-hydroxyl group of ADP-ribose on ADP-ribosylated protein substrates and the C-terminus of Ub. SCF^FBS2/FBXO6^ first binds to the *N*-glycan on the glycoprotein, forming a complex with ARIH1 to polyubiquitinate (via oxyester bonding) the 6-hydroxyl group of the *N*-acetylglucosamine (GlcNAc) residue that remains on the asparagine residue after endo-*N*-acytylglucosaminidase cleaves the *N*-glycan from the glycoprotein. (**B**) Ubiquitination of non-proteinaceous molecules. RNF213 conjugates Ub to the lipid A moiety of lipopolysaccharide (LPS) on infecting bacteria via an oxyester bond (precise attachment site unknown). Tul1 conjugates Ub to the amino group of phosphatidylethanolamine (PE) via an amide bond. HOIL-1 conjugates Ub (via an oxyester bond) to the C6-hydroxyl group of less-branched glucose polymers such as polyglucosan, which are associated with HOIP and SHAPIN components of LUBAC. DTX3L/DTX3 conjugates Ub to the 3ʹ-hydroxyl group of ssDNA/ssRNA via an oxyester bond. ZNFX1, activated by long-chain ssRNA, conjugates Ub to the 2ʹ-hydroxyl group on the internal ribose moiety of ssRNA via an oxyester bond. The conjugation of Ub to synthetic compounds via amide bonds has also been reported: RNF19 conjugates Ub to the azetidine nitrogen of BRD1732, whereas HUWE1 conjugates Ub to the primary amino groups of BI-8622 and BI-8626. (**C**) The concept proposed in this review.

Building on these findings, recent studies have revealed that ubiquitination extends beyond amino acid side chains. Ub can be conjugated to other PTM targets, most notably glycans and ADP-ribose (ADPr) moieties, and to free non-proteinaceous biomolecules such as lipids, sugars, metabolites, nucleic acids, and synthetic small-molecule compounds. Collectively, these discoveries reframe the identity of Ub as a versatile modifier capable of acting across the full breadth of cellular biochemistry rather than merely a protein-modifying tab, as described in recent excellent review articles [10–12,30–32]. In this mini-review, we focus on ubiquitination events targeting non-amino-acid substrates and provide an update on the latest advances in illuminating the expanding functional landscape of Ub (Figure 2).

## Ubiquitination of lipopolysaccharide and phospholipids

The first example of non-proteinaceous ubiquitination was discovered in the context of intracellular immunity [33]. This pioneering study demonstrated the ubiquitination of bacterial LPS by the host E3 ligase RING finger protein (RNF) 213, also known as Mysterin, the largest E3 ligase encoded in the human genome [33]. Subsequent studies have begun to clarify both the activation mechanism of RNF213 and the bacterial strategies that counteract this host defense pathway.

Upon *Salmonella* invasion, RNF213 ubiquitinates the lipid A moiety of LPS, a process that requires a specific zinc finger architecture (known as the RZ finger) together with an ATP-binding domain. This ubiquitination primarily requires Ub-conjugating enzyme E2 L3 (UBE2L3). The ubiquitination of LPS serves as a platform for the recruitment of LUBAC, resulting in the formation of multiple Ub coats composed of K63 and M1 linkages surrounding the bacteria cells. These Ub structures recruit autophagy receptors and trigger the autophagic sequestration of the invading bacteria, a process named xenophagy [34]. Although the precise site of Ub attachment within lipid A remains unknown, this linkage is sensitive to alkaline hydrolysis, which suggests the formation of an oxyester bond [33]. RNF213 activity is reportedly also stimulated by ATP binding, which functions as a cytosolic danger signal during infection [35]. Pathogenic bacteria have evolved mechanisms to counteract this system. For example, *Burkholderia* species secrete TssM, a potent Ub esterase that directly removes RNF213-conjugated Ub from LPS [36]. *Shigella* uses an alternative strategy, whereby the effector IpaH1.4 binds to the RING domain of RNF213 and ubiquitinates the ligase, thereby promoting its proteasomal degradation [37].

In addition to LPS, intracellular phospholipids, primarily PE, have been reported to undergo ubiquitination. PE ubiquitination is conserved from yeast to humans and occurs mainly on endosomal and lysosomal membranes. For ubiquitination, the C-terminal glycine residue of Ub is conjugated to the amino group of PE under the control of specific enzymatic systems; namely, Ub-conjugating enzymes 4/5 (Ubc4/5) and transmembrane Ub ligase 1 (Tul1). This process is reversed by the DUB Doa4 in yeast. Lipid ubiquitination is enhanced under stress conditions such as nutrient starvation, and may promote the recruitment of endosomal sorting complexes required for transport (ESCRT) components to membranes. Notably, Ub-like proteins such as neural precursor cell expressed, developmentally down-regulated 8 (NEDD8) and interferon-stimulated gene 15 (ISG15) can also be attached to lipids, suggesting that members of the Ub family share the ability to modify lipids [38].

## Ubiquitination of sugars, metabolites, and glycans

Most sugar chains are synthesized in organelles such as the endoplasmic reticulum and the Golgi apparatus. Glycogen is a notable exception in that it is synthesized in the cytosol from glucose-derived UDP-glucose and serves as a primary energy storage polymer. Glycogen comprises branched sugar chains. However, polyglucosan with reduced branching is insoluble, and the resulting precipitates (named polyglucosan bodies) accumulate in tissues such as the myocardium and brain of patients lacking the LUBAC component HOIL-1, resulting in cardiomyopathy and heart failure [39]. Kelsall et al. identified polyglucosan bodies in the brains of mice expressing catalytically inactive HOIL-1 and proposed that this LUBAC domponent might ubiquitinate not only serine and threonine residues but also polyglucosan itself in collaboration with UBE2L3 [40]. Subsequently, they found that the two other LUBAC components HOIP and SHARPIN preferentially bind to heptamaltose rather than to branched glycogen. HOIL-1 conjugates Ub to the C6 hydroxyl group of glucose and transfers the preformed Ub chains *en bloc*, thereby generating an autophagy-targeting degradation signal [40]. The histidine-510 residue of HOIL-1, which is essential for oxyester-linked ubiquitination, has been reported to suppress ubiquitination at the lysine residues of target substrates [41]. Notably, the ubiquitination of C6 hydroxyl groups is not restricted to glucose, suggesting that a broad range of sugars may serve as Ub acceptors [41]. Among the 14 known RBR E3 ligases, RNF216, which catalyzes canonical lysine ubiquitination, has been shown to ubiquitinate sugars such as maltose *in vitro* [42]. Recently, Jochem et al. have developed an innovative method designated ‘Non-Protein Ub-clipping (NoPro-clipping)’ to analyze non-proteinaceous ubiquitinated substrates that are difficult to detect using conventional Ub research approaches [43]. The NoPro-clipping method labels substrates using two bacterial-derived enzymes, a Ub clippase (either Lb^pro*^ or BpJOS) and sortase. Target biomolecules labeled with ClipTag were subsequently detected based on liquid chromatography–tandem mass spectrometry analysis, leading to the discovery of ubiquitinated metabolites, such as glycogen, glycerol, and spermine, in living organisms. Notably, during the early stages of fasting-induced glycogen depletion in mice, approximately 1% of all Ub was attached to glycogen in the liver. Furthermore, these authors suggested that the ubiquitination of glycogen is involved not only in the elimination of abnormal aggregates observed in glycogen storage diseases but also plays a role in physiological glycogen metabolism, including lysosomal degradation during fasting.

With regards to protein glycosylation, although most *N*-glycosylated proteins are found in organelles of the secretory pathway and on the cell surface, certain glycoprotein-related enzymes reside in the cytosol. Loss-of-function mutations in *NGLY1*, which encodes a cytosolic de-*N*-glycosylating enzyme, cause NGLY1 deficiency (a rare multisystem congenital disorder), underlighting the physiological importance of cytosolic deglycosylation [44]. A glycan-recognizing Ub ligase, SCF^FBS2/FBXO6^, which mediates the ubiquitylation of ERAD substrates, has been implicated in the pathogenesis of NGLY1 deficiency [45]. Nuclear factor erythroid 2-related factor 1(Nrf1/NFE2L1) is a heavily *N*-glycosylated protein and an intrinsic ERAD substrate. However, when proteasome activity declines, Nrf1 escapes degradation, translocates to the nucleus, and drives the transcriptional induction of proteasome subunit genes [46]. NGLY1 activates Nrf1 by concurrently removing its *N*-glycans and editing specific amino acid residues (converting asparagine to aspartic acid) [47,48]. In the absence of NGLY1 activity, SCF^FBS2^ binds to the *N*-glycans of Nrf1. This substrate engagement recruits Ariadne RBR E3 Ub protein ligase 1 (ARIH1; an RBR-type E3 ligase) to NEDDylated Cullin 1 (CUL1), which subsequently ubiquitylates Nrf1 in conjunction with UBE2L3 [49]. Because the activity of endo-β-*N*-acetylglucosaminidase (ENGASE, which generates *N*-acetylglucosamine (GlcNAc) from *N*-glycans) is also required for this reaction, the Ub chains are surmised to be attached to the trimmed GlcNAc residues. Specifically, ARIH1 forms atypical Ub chains at two distinct sites: the C6 hydroxyl group of GlcNAc, and the serine and threonine residues within *N*-glycosylation sequons. This ubiquitination requires the binding of SCF^FBS2^ to a neighboring *N*-glycans [49]. Although the accumulation of abnormally ubiquitinated Nrf1is known to cause proteasomal dysfunction and cellular damage, but the precise mechanism remains unknown. Unlike HOIL-1, ARIH1 also ubiquitinates lysine residues [50], and in the case of Nrf1, oxyester-linked ubiquitination is postulated to be favored owing to the paucity of lysine residues near *N*-glycosylation sites.

## Ubiquitination of ADP-ribose, and nucleic acids

The human DELTEX (DTX) family of E3 ligases (comprising DTX1, DTX2, DTX3, DTX3L, and DTX4) has emerged as key mediators of the ubiquitination onto ADPr moieties in proteins and nucleic acids, acting in conjugation with ADP-ribosyl transferases, in particular the poly(ADPr) polymerases (PARPs) [51]. It has been found that DTX enzymes transfer Ub to the 3′-hydroxyl group in the adenine-proximal fragment of ADPr or to ADP-ribosylated protein in conjugation with UBE2D, and Ub-specific protease 2 (USP2) or SARS-CoV2-PLpro can remove the Ub molecules [52]. Recent studies have shown that DTX2 and PARP7 promote the ADP-ribosylation-dependent ubiquitination of androgen receptor and aryl hydrocarbon receptor, thereby targeting them for proteasomal degradation [53,54]. This mechanism, termed ADP-ribosylation-dependent degradation, represents an emerging paradigm for post-translational control of transcription factor activity, and potentially other cellular processes. Furthermore, beyond functioning as a standalone signal, ubiquitinated ADPr (ADPr-Ub) can serve as a signal for Ub chain elongation, as the E3 ligases RNF114 and its paralogs (RNF125, RNF138, and RNF166) have been shown to extend K11-linked Ub chains by detecting ADPr-Ub conjugates during DNA damage response [55–57]. In addition, the ubiquitination of ADPr has been shown to contribute to protein stability. For example, tankyrase, a poly-ADPr transferase, conjugates poly-ADPr to itself, resulting in its ubiquitination by poly-ADPr-binding E3 ligase RNF146 and subsequent proteasomal degradation. Counteracting this degradation, tankyrase is mono-ubiquitinated by DTX2 and DTX3 and then polyubiquitinated by RNF114 and RNF166, thereby suppressing RNF146-mediated degradation [58].

In addition to their role in PTM ubiquitination, some DTX enzymes have been shown to conjugate Ub to nucleic acids. Zhu et al. found that DTX3L and DTX2 attached Ub to ADP-ribosylated single-stranded nucleic acids (ssDNA/ssRNA) [59]. Subsequent studies showed that DTX3L and DTX3 directly attach Ub to the 3ʹ-hydroxyl group of ssDNA/ssRNA [60,61]. This reaction requires both the RING domain and the DTC domain of DTX3L, with the DTC domain mediating nucleic acid binding. Similar to ubiquitination of ADPr on proteins, nucleic acid ubiquitination is reversible, with the modification removable by DUBs. In addition to DTX enzymes, zinc finger NFX1-type containing 1 (ZNFX1) functions as an RNA-associated E3 ligase together with an SF1-type RNA helicase that couples RNA sensing to Ub signaling. Upon recognizing long ssRNAs in an ATP-dependent manner, ZNFX1 undergoes robust auto-ubiquitination and promotes the formation of higher-order protein assemblies. Grabarczyk et al. have reported that ZNFX1 directly ubiquitinates the 2′-hydroxyl group on the internal ribose of RNA, thereby promoting the formation of dense Ub-coated ribonucleoprotein particles [62]. Conversely, Squair et al. have argued that rather than undergoing direct ubiquitination, the associated RNA is instead ‘quarantined’ within self-propagating ZNFX1 aggregates [63]. However, despite these differing observations, the authors of both studies agree that this RNA-activated ubiquitination machinery is essential for regulating RNA homeostasis, dampening stress responses during interferon signaling, and promoting cell survival [62,63].

## Ubiquitination of small-molecule compounds

Recent studies have revealed that synthetic small-molecule compounds can serve as direct substrates for ubiquitination, thereby expanding the known repertoire of Ub acceptors beyond endogenous biomolecules. BRD1732, a synthetic compound derived from a diversity-oriented synthesis library, is ubiquitinated within cells [64]. This modification is stereospecifically catalyzed by RNF19A and RNF19B in conjunction with UBE2L3, resulting in the covalent attachment of Ub C-terminus to the azetidine nitrogen (a second amine) of BRD1732. Notably, the accumulation of Ub-BRD1732 causes broad inhibition of the Ub proteasome system, suggesting that the ubiquitination of small-molecule compounds can have far-reaching consequences for cellular proteostasis. In a parallel line of investigation, the HECT-type E3 ligase HUWE1 was shown to ubiquitinate the small-molecule compounds BI-8622 and BI-8626, which were previously characterized as HUWE1 inhibitors [65]. In this study, Ub was conjugated to the primary amino groups of BI-8622 and BI-8626. *In vitro* experiments demonstrated selectivity for HUWE1, and the compounds competed with protein substrates for modification. Both UBE2L3 and UBE2D3 are involved in the ubiquitination of BI-8622 and BI-8626, suggesting that this reaction is not limited to a specific E2 enzyme.

Taken together, these reports establish small-molecule compounds bearing amine groups as a new class of non-proteinaceous substrates for ubiquitination. The finding that both primary and secondary amines serve as Ub acceptors raises the intriguing possibility that a broad range of amine-containing compounds may be subject to ubiquitination *in vivo*.

## Concluding remarks

The studies reviewed herein collectively demonstrate that ubiquitination is a far more chemically versatile modification than implied by its canonical definition. Across all the non-proteinaceous substrates discussed—LPS, lipids, sugars, metabolites, ADPr, nucleic acids, and small molecule compounds— a common chemical principle emerges. Presumably, Ub is evenly incorporated into the ε-amino group of lysine and various nucleophilic groups, including hydroxyl and thiol groups (via oxyester and thioester bonds) and amine (via isopeptide-like amide bonds) groups. The reversibility of several of these modifications by DUBs indicates that non-canonical ubiquitination is actively regulated, although the functional significance may vary depending on the context, reflecting either a *bona fide* signaling cycle or protective removal of potentially deleterious modifications.

Notably, many of the E3 ligases that mediate non-canonical ubiquitination (RNF213, HOIL-1, ARIH1, DTX3L, ZNFX1, RNF19, and HUWE1) are well-characterized enzymes of the Ub system. This suggests that the capacity for targeting non-proteinaceous substrates is more widespread among E3 ligases than is currently appreciated. In support of this supposition, E2 enzymes (e.g. UBE2J2 and UBE2Q) capable of conjugating Ub to the hydroxyl groups of serine, threonine, glycerol, and glucose have been identified *in vitro* [66]. Further studies to clarify the relationship between E3 ligases and non-canonical substrates are anticipated. Interestingly, ARIH1 unbiasedly transfers Ub to lysine, serine, threonine, and non-proteinaceous acceptors when they are equally close to E3 ligases. This suggests that the structural distance between the Ub acceptor groups and the catalytic core of E3 ligase mainly determines the resulting modification form. Given the historical context of this PTM and the limitations of detection systems, the ubiquitination of lysine may have been considered the conventional form. As detection methods improve and systematic screening becomes more feasible, new classes of ubiquitinated substrates will continue to emerge in future studies. Indeed, the NoPro-clipping method has contributed to revealing the unexpectedly high levels of glycogen ubiquitinated by LUBAC present *in vivo*, as well as the potential production of diverse ubiquitinated metabolites, although the cognate E3 ligases have yet to be identified. Taken together, these findings invite a fundamental reconceptualization of Ub as a versatile chemical modifier whose substrates span the entire realm of cellular biochemistry. In concert with investigations on the ubiquitination of novel non-proteinaceous substrates, the physiological significance of this PTM is expected to be elucidated in the next decade.

## Perspectives

- Ubiquitination has long been regarded as a protein-specific modification. However, the findings that Ub can be conjugated to other biomolecules, such as lipids, sugars, metabolites, ADP-ribose, and nucleic acids, as well as chemicals fundamentally expands our understanding of its role as a general modifier in cell biology.
- Recent biochemical and structural studies have revealed the enzymatic mechanisms underlying non-canonical ubiquitination. However, the physiological roles of most non-proteinaceous substrates remain to be elucidated.
- Advances in chemical proteomics, together with the development of tools for detecting oxyester-linked ubiquitination *in vivo*, are essential to fully elucidate the scope and biological significance of this emerging modification landscape.

## Open Access

Open access for this article is provided via our Subscribe to Open programme, made possible through the support of our subscribing institutions. Learn more here: https://portlandpress.com/pages/open_access_options_and_prices#conditional.

## Abbreviations

ADPr: ADP-ribose
ARIH1: Ariadne RBR E3 Ub protein ligase 1
DUB: deubiquitinating enzyme
ENGASE: endo-β-*N*-acetylgulucosaminidase
ERAD: endoplasmic reticulum-associated degradation
ESCRT: endosomal sorting complexes required for transport
GlcNAc: *N*-acetylglucosamine
HECT: homologous to the E6-AP carboxyl terminus
IRAK: interleukin receptor-associated kinase
LPS: lipopolysaccharide
LUBAC: inear Ub chain assembly complex
NF-κB: nuclear factor kappa B
NoPro-clipping: Non-Protein Ub-clipping
Nrf1/NFE2L1: nuclear factor erythroid 2-related factor 1
PE: phosphatidylethanolamine
PTM: post-translational modification
RBR: RING-in-between-RING
RING: really interesting new gene
RNF: RING finger protein
Ub: ubiquitin
UBE2L3: Ub-conjugating enzyme E2 L3
ZNFX1: zinc finger NFX1-type containing 1
